# A conceptual model for automating spatial network analysis

**DOI:** 10.1111/tgis.12855

**Published:** 2021-10-25

**Authors:** Simon Scheider, Tom de Jong

**Affiliations:** ^1^ Department of Human Geography and Spatial Planning Utrecht University Utrecht the Netherlands; ^2^ Department of Logistics Stellenbosch University Stellenbosch South Africa

## Abstract

Spatial network analysis is a collection of methods for measuring accessibility potentials as well as for analyzing flows over transport networks. Though it has been part of the practice of geographic information systems for a long time, designing network analytical workflows still requires a considerable amount of expertise. In principle, artificial intelligence methods for workflow synthesis could be used to automate this task. This would improve the (re)usability of analytic resources. However, though underlying graph algorithms are well understood, we still lack a conceptual model that captures the required methodological know‐how. The reason is that in practice this know‐how goes beyond graph theory to a significant extent. In this article we suggest interpreting spatial networks in terms of quantified relations between spatial objects, where both the objects themselves and their relations can be quantified in an extensive or an intensive manner. Using this model, it becomes possible to effectively organize data sources and network functions towards common analytical goals for answering questions. We tested our model on 12 analytical tasks, and evaluated automatically synthesized workflows with network experts. Results show that standard data models are insufficient for answering questions, and that our model adds information crucial for understanding spatial network functionality.

## 1 INTRODUCTION

Computational models of spatial networks for geographic information systems (GIS) have been known for a long time (Sutton, 1998). They are frequently used in applications such as spatial planning (Geertman, de Jong, & Wessels, 2003), transport analysis (Thill, 2000), supply infrastructures, and the analysis of flows (Curry, 1972, cf. Miller & Shaw, 2001, for an overview). Corresponding functions are nowadays implemented in many GIS software tools, such as ArcGIS Network Analyst (https://www.esri.com/en‐us/arcgis/products/arcgis‐network‐analyst/overview), as well as in Web APIs and geo‐services (https://developer.here.com/).

Yet, despite the ubiquity of technical resources, answering questions about spatial networks still requires organizing analytic functionality into workflows, and the latter presupposes a considerable amount of expertise. Suppose our task is to assess the accessibility and distribution of transport flows within a road network. Could ArcGIS’s *service area* tool (https://pro.arcgis.com/en/pro‐app/latest/help/analysis/networks/service‐area‐analysis‐layer.htm) be used for this task, or rather a different one? And is a road network data set sufficient, or do we need travel statistics as well? It is clear that while such tasks are of relevance for many data scientists, manual identification of functions and data is a time‐consuming process (Scheider & Tomko, 2016), and manual composition of workflows remains a non‐trivial craft.

To address this challenge, *program synthesis algorithms* were developed in (symbolic) artificial intelligence (AI)^1^ (Naujokat, Lamprecht, & Steffen, 2011). They provide a way to automate this task, allowing analysts to loosely specify workflows without knowing the details about available resources (Kasalica & Lamprecht, 2020b). These algorithms have predecessors in geographical information service composition (Lutz, 2007), but go beyond by searching through the composition space of functions described by an information ontology, in order to satisfy a given task specification (Lamprecht, Naujokat, Margaria, & Steffen, 2010). To automate spatial network analysis, the main challenge lies in finding the appropriate semantic constraints for both task specifications and function descriptions (Kruiger et al., 2021).

Yet geographic information science (GIScience) has struggled to come up with a model that is able to capture the semantic constraints implied by this practice (see Section 2). The difficulty seems to lie in a frequent confusion of *networks as concepts* used in geographic practice, with *networks as data models* implemented in particular information systems (Kuhn & Ballatore, 2015). Network data models are usually understood as *embedded graphs* (Scheider & Kuhn, 2008), where vertices are embedded as points in Euclidean space allowing us to assess metric distances. While sufficient for implementing network procedures, this model seems to disregard important concepts needed to analyze spatial networks, and in consequence, fails to capture underlying analytical tasks. To illustrate, suppose our goal is to assess the effect of football games on traffic load on the streets, caused by football fans traveling to their respective clubs. How could a graph model be used to specify the task of determining flows of fans from residential areas to clubs based on the numbers of residents and their distances to clubs? There is no concept in embedded graphs that would allow us to distinguish numbers from ratios on nodes or flows from distances on edges. Another sub‐task is to assign flows to particular paths on a road network to assess the traffic caused by fans. To handle this problem, different kinds of weights for different kinds of edges need to be distinguished, yet we currently lack a theory that makes such distinctions. Sticking to graph‐theoretic terms seems to merely transfer the problem to the semantics of graph labels, or of edge and node labels (Kanjilal & Schneider, 2010).

We therefore argue that the concepts underlying spatial network data models need to go beyond embedded graphs. An explicit model of these concepts would help us better understand not only what kind of information a spatial network contains, and which questions can therefore be answered with it (Scheider et al., 2021), but also what kinds of analyses are possible. This leads to automating the analysis process itself. To address this goal, we argue that spatial networks should be conceived in terms of *core concepts of spatial information* (Kuhn, 2012), which implies important restrictions on the applicability of functions. More precisely, we consider networks as quantified relations between spatial objects,^2^ where both object and relational qualities can be considered *extensive or intensive* (Scheider & Huisjes, 2019). Spatially extensive measures are *additive* with respect to the spatial extent of their controlling objects, whereas intensive measures are not additive in this sense. Consider, for example, the potential of football fans living in districts of a city. These potentials add up when merging underlying districts, as opposed to the distance to the city center. Notice that both potential and distance measures are required for estimating travel flow in our example above, and more generally, to model spatial network analysis (see Section 4.2).

We argue that this new model, by its very simplicity, can go a long way towards clearing a pathway through the jungle of available functionality and corresponding network tasks. We focus on the following questions:

1. How can spatial network tasks be specified in terms of core concepts and extensivity, to assess the suitability of resources?
2. To what extent can network functionality be distinguished in terms of concept transformations?
3. What is the quality of automatically synthesized workflows that are based on such concepts?

Note that in this article, spatial network analysis is not a method, but an object of investigation. Correspondingly, we are not targeting empirical questions about spatial networks, as usually intended by GIS analysts. Instead, our study is about conceptual modeling (Guarino, Guizzardi, & Mylopoulos, 2020) of geographic information, and a network analysis scenario serves merely as our empirical basis. Even though our goal is to distinguish network concepts from other kinds of concepts relevant in GIS, we are aware that the underlying functions always form an integrated whole in practice. Correspondingly, our model feeds into a more general geographic information ontology (Scheider et al., 2020). Our goal is a *lightweight type system* that is able to model the part of this practice needed to compose workflows for answering questions (Scheider et al., 2021). In the following, we start with a review of spatial network theory and corresponding conceptual models (Section 2), before giving an overview of our methodological approach (Section 3). Our own conceptual model is developed in Section 4, and is then used to introduce computational signatures for spatial network functions (Section 5), as well as to specify 12 spatial network tasks in an application scenario (Section 6). Finally, we evaluate our model by automatically synthesizing workflows for each scenario task and by assessing their quality (Section 7).

## 2 RELATED WORK

If we look at current standard textbooks on GIS, spatial networks seem to play only a minor role (Burrough, McDonnell, & Lloyd, 2015; Chrisman, 2002; Heywood, Cornelius, & Carver, 2010; Longley et al., 2015). Yet the relevance of spatial networks for geo‐spatial analysis has been known to geographers since the rise of quantitative methods in the second half of the twentieth century. It is insightful to take a look at the history of spatial network related concepts, which runs in parallel to the change of research paradigms within geography and GIScience. Furthermore, we review recent work on geospatial semantics as a basis for modeling spatial network concepts.

### 2.1 Spatial network analysis

Peter Haggett and Richard Chorley’s book (Haggett & Chorley, 1969) provides an early integrated view on passive (drainage networks) and active transportation networks (e.g. roads). In this text, graph theory plays a minor part, including definitions of trees and circular graphs, as well as shortest path algorithms. Beyond graphs, the authors focus their discussion on *flow* networks versus *barrier* networks; relations of channel *order numbers*, *flow* and *lengths* in drainage networks; geometric *shapes, densities and orientations* of networks; the relation between *distance*, *flow* and *efficiency/costs* of networks, relating to Christaller’s optimal settlement system (Christaller, 1933), as well as network *change over time*. Furthermore, optimization methods include not only shortest path algorithms, but also *districting* and problems of *regionalization* (how to divide space into tessellated regions using networks).

In the 1970s and 1980s, when GIS evolved, human geographers discovered the powerful concept of a *potential* in geographic space (Rich, 1980). This is related to the idea of *accessibility*, which combines the concept of *distance* with the *utility* of activities that can be performed at the destinations in a network (Moseley, 1979; Ingram, 1971). Accessibility allows us to assess a *potential interaction* (Masser & Brown, 1977) of *numbers of people or amounts of goods* between *places*, in analogy to a gravity model (Batty, 1976; Curry, 1972; Wilson, 1974). These methods have become essential tools of spatial planning with GIS (Geertman & Ritsema van Eck, 1995; Jong & Ritsema van Eck, 1996). Besides path algorithms, Ritsema van Eck (1993) identified *zoning*, *districting* and *origin–destination matrix* methods as essential for spatial network analysis in GIS.

Research on spatial network models and GIS during the 1990s, in contrast, focused less on conceptual or methodological issues, and more on network data models that would allow integration of transport science functionality into GIS databases (Miller & Shaw, 2001; Sutton, 1998; Thill, 2000). These systems were called GIS‐T, and researchers were mainly concerned with how data structures and algorithms for transportation research could best be integrated within a GIS infrastructure. This “structural” view of networks continues to the present day, though the focus has shifted from implementation models to formal models that would support efficient design of databases across software environments (Kanjilal & Schneider, 2010; Qi, Zhang, & Schneider, 2016), as well as efficient querying of network data, including graph databases (Güting, 1994) and moving objects on networks (Güting, De Almeida, & Ding, 2006). Other authors have focused on network complexity measures for spatial graphs (Arlinghaus, Arlinghaus, & Harary, 2002; Jiang & Claramunt, 2004). The latter approach, however, largely abstracts from the conceptual basis of network analysis in geography.

### 2.2 Networks as core concepts of spatial information

What kind of semantics should be adopted to model spatial networks as concepts? Some researchers have been investigating transport networks from the viewpoint of environmental cognition, such as wayfinding activities and affordances (Winter, 2002; Scheider & Kuhn, 2010, 2008). A more general, transdisciplinary account of networks was given by Kuhn in terms of the *core concepts of spatial information* (Kuhn, 2012). On this account, networks are one of a range of concepts needed for interpreting the environment and for reasoning with GIS. These concepts constitute conceptual “lenses” through which the environment can be studied independently of technical representations (Allen et al., 2016; Kuhn & Ballatore, 2015). Besides the base concept of *location*, allowing for metric distance assessments in space, Kuhn distinguished the following content concepts, which we interpret here in a broader research context:

- *Fields* are understood as continuous functions (Galton, 2004) whose domain is time and location, and whose range may be any kind of measurable quality. Temperature fields are a prime example.
- *Objects* are understood as functions from time to locations and qualities (Galton, 2004). Objects are distinct from fields and events in the sense that they have an identity and that they are fully localized in each moment of their existence. We assume that objects include both bona fide (perceivable) and fiat (conventional) boundaries, as in the case of administrative units.
- *Events* are understood as entities that, besides having identity and having qualities like objects, happen during some temporal interval. Earthquakes, which have a time, a location, and a magnitude, are a prime example.
- *Networks* are quantified relations between objects, that is, functions from pairs of objects to some quality. Networks measure a relationship between objects. Kuhn (2012) distinguished *link networks* which connect objects in a qualitative way (e.g. friendship, treaty or business relation) from *path networks*, which can measure flows or paths between objects. Similar distinctions can be drawn in our model.

We believe that geo‐analytical tasks, and network analysis in particular, can only be understood when modeling these concepts in combination, because they depend on each other. Yet, so far, computational models of core concepts have not taken networks into focus (Kuhn & Ballatore, 2015). Furthermore, it is an open question how core concepts combine with other semantic concepts needed for geographic analysis (Scheider et al., 2020). Our model of spatial networks was designed to reflect precisely this underlying practice.

### 2.3 Ontologies for geo‐analytic workflow synthesis

Automated workflow composition first appears in the context of geographical information web processing services (Yue et al., 2007). However, its effectiveness mainly depends on the quality of the ontology used to describe the information resources (Hofer et al., 2017). As recognized early on (Albrecht, 1998; Giordano et al., 1994), this includes the need for generalized taxonomies of GIS that focus on functionality rather than technicalities. The main difficulty seems to lie in the fact that analytical concepts are not fully reflected in data types, and thus can occur in various syntactical variations. In Scheider et al. (2020), we have therefore suggested an OWL^3^ ontology of types of core concepts that can occur in combination with measurement levels and data types, to serve as a method for *reasoning about* GIS workflows and geo‐analytical tasks. Based on this work, there have been recent attempts at automating GIS workflow synthesis for tasks that are not network related (Kruiger et al., 2021). Computationally, this approach is based on loose programming, that is, the sequencing of functions satisfying task constraints specified over an ontology with some temporal logic (Lamprecht, Naujokat, Margaria, & Steffen, 2010) (see Section 7.1). To handle spatial network analysis tasks in the same manner, network concepts need to be *combined* with other core concepts. Yet, formal models of the role that networks play in this respect are lacking. We also do not know of any studies about modeling network functionality with the goal of automating geo‐analytical tasks. This gap is addressed in the current article.

## 3 METHODOLOGY AND APPROACH

In this section we explain the steps taken towards developing and testing a conceptual model of spatial network analysis. Empirically, our study is based on a network analysis scenario: the analysis of football clubs and their fans in the Netherlands, as outlined below. This scenario gives us a way to explore core tasks of spatial network analysis as a basis for developing our model (Grüninger & Fox, 1995). Furthermore, to evaluate our model, we manually generated expert‐level workflows for these tasks, and compared them with workflows automatically synthesized using our conceptual model.

### 3.1 Network analysis scenario and task design

The following scenario was selected based on whether it captures precisely those practices that distinguish spatial network analysis from other types of spatial analysis. This mainly includes the capabilities of handling spatial interaction data, going beyond geometrical GIS models that focus on topological relations and distances. Dejonghe, Van Hoof, and Kemmeren (2006) published a book on professional football clubs and their fan base in the Netherlands. One of the data sets they used is the 2003 nationwide complete list of the number of seasonal ticket holders by football club and by municipality. Football fans in the Netherlands are usually season ticket holders, and as such form regular transport flows when traveling to their clubs.

**FIGURE 1:**
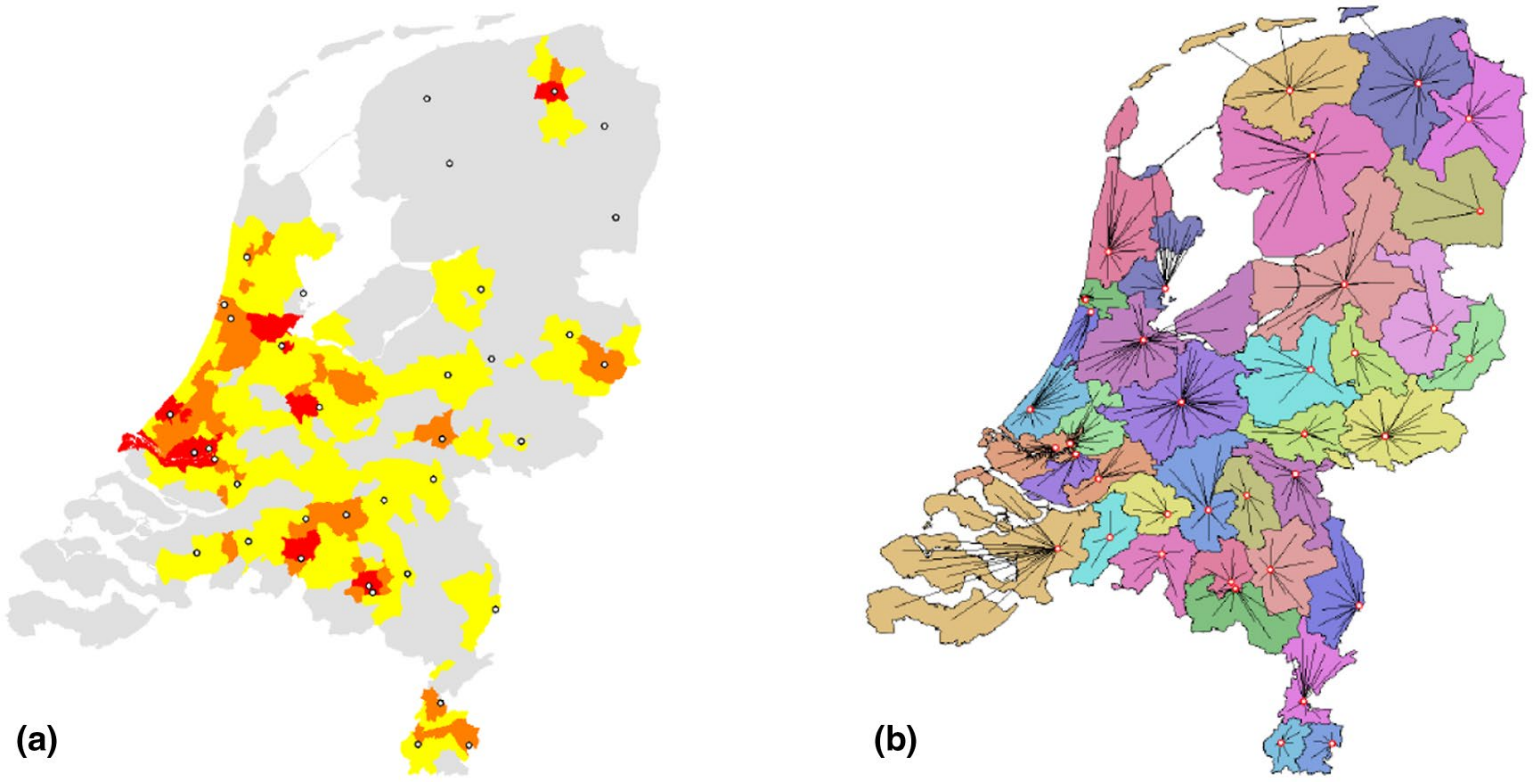
(a) Fan potential and (b) club accessibility. (a) threshold distance map, showing the travel distance to reach a given number of fans on a scale from red (5,000 tickets within a five‐minute drive time) via orange (5,000 within 10 min) to yellow (5,000 within 15 min). Stadiums are shown as points. (b) Catchment areas are drawn in different colors for each club and the superimposed allocation lines indicate the closest club for each municipality. The analysis generates both shortest distances to closest club and information about this club

We assume an analyst plans a follow‐up GIS study exploring spatial interaction of the fan base at a municipal scale. Suppose he or she is given municipal data about population numbers, football clubs (within municipalities), a road network, and some data about fan (ticket) statistics. Using these data, the analyst can answer various network‐related questions. In total, we formulated *12 different workflow tasks* that cover major forms of analysis (Section 6). For illustration purposes, we explain the first three examples:

1. What is the suitability of municipalities (e.g. as a place for a new stadium) in terms of the fan potential reachable within a certain distance?
2. What is the suitability of municipalities in terms of the minimal travel distance to reach a certain number of football fans?
3. What is the accessibility of football clubs for people living in municipalities?

Workflows to answer the first two questions can be found using *threshold distance/amount analysis*. For example (*Task 1*), we can assess the minimal distance that needs to be traveled to reach a threshold number of potential fans, which generates a map of municipal travel times (Figure 1a). Alternatively (*Task 2*), one could assess the number of football fans reachable within a threshold distance (not shown here). Answers to the third question can be found by generating a map of *catchment areas* (*Task 3*), where each municipality is assigned to its nearest club according to some club capacity. This results in a map like in Figure 1b, where the smaller the distance, the more accessible clubs are. The data can be used to do accessibility statistics, revealing, for example, that over 77% of ticket holders live near at least one club within a 15‐minute drive.

### 3.2 Expert‐level workflow design

**FIGURE 2:**
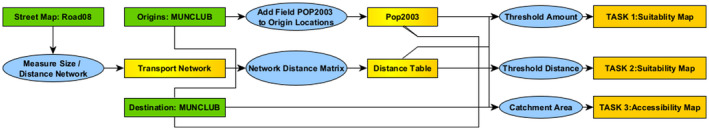
Expert workflow implementing Tasks 1, 2 and 3 in Flowmap. Ellipses denote computational steps, rectangles denote data sets. We have generated such expert solutions for every task (not shown here because of lack of space); see Section 7

Once analytical tasks were formulated, we designed workflows manually as a basis for developing and evaluating our model. We were interested in understanding how experts choose and organize software tools into a workflow graph which generates valid answer maps. The answers were computed and illustrated using Flowmap, which is a software designed to handle spatial interaction (http://flowmap.geo.uu.nl/). Some of this functionality can also be found in other GIS software, such as ArcGIS Network Analyst (https://www.esri.com/en‐us/arcgis/products/arcgis‐network‐analyst). Example workflows for answering Tasks 1,2 and 3 can be seen in Figure 2.

To computationally solve these three tasks, we first need to measure the length of road segments (or their travel impedance) using street data. Then we need to turn the latter into a transport network (graph), by taking segment ends as intersections. This also includes checking segment topology. Origin and destination locations (municipalities) together with the transport network then need to be fed into a distance matrix function to compute a matrix of shortest paths between municipalities (including also the “last mile” feedlinks from origins and destinations to the closest network intersection). The distance matrix together with the origin (destination) locations including their capacity (demand) then feed into either a threshold or a catchment area function, to produce either a suitability or an accessibility map. *Parameters* (not shown here) are the use of travel speed for computing distances as travel time, as well as the choice of threshold distance or amount.

### 3.3 Conceptual modeling and workflow synthesis study

The goal of our investigation is to learn how to produce workflows comparable to the examples above in an automated manner, given just the task descriptions and the starting data. Since these computational steps are implicit in the task, they need to be figured out automatically. This is done based on some conceptual model that can be used to describe the task, the data and the computational functions. We develop such a model in Section 4 in the form of an ontology. We used this ontology to describe typical spatial network functions as *transformations of concepts* in our model. This means we described functions in terms of their input/output types, resulting in a *type signature* in Section 5. Furthermore, we *specified the 12 analytical tasks* in terms of concept transformations using the same types (Section 6).

The conceptual model, together with the task specification and the function type signatures were then fed into a *loose programming algorithm*. The latter searches for ontologically consistent sequences of function applications of increasing complexity that satisfy a given task description (Lamprecht, Naujokat, Margaria, & Steffen, 2010). As explained in Section 7, we evaluated synthesized workflows based on expert assessments.

## 4 A CONCEPTUAL MODEL OF SPATIAL NETWORKS

The model introduced in this section is less about computation, and more on the level of *thinking in GIS*. Thinking happens in parallel to computation by interpreting the computational products in terms of concepts (Guarino, Guizzardi, & Mylopoulos, 2020). In a nutshell, we suggest regarding spatial networks as quantified relations between objects embedded in a metric space, such that both objects and their relations can be quantified in a spatially extensive or intensive manner. This model is used to formulate analytical tasks and to guide the composition of workflows.

### 4.1 Spatial networks as quantified relations

One way to think of core concepts of spatial information (Kuhn, 2012) is in terms of particular kinds of *relations* in the sense of relational algebra^4^ (Codd, 1979). For example, information about a spatial field can be regarded as a relation between locations and some quality (“at this location, the temperature is $15^{\circ }$C”), and information about objects as a relation between object identifiers and object qualities (“this building has a height of 10 m”). In the first case, locations form the primary key, in the second case, object identifiers serve as the primary key, while qualities are foreign keys in all cases. We call such relations *unary qualities*, because *the measured quality is controlled by a single entity*. A spatial network, in contrast, captures the idea of a relation with a *composite key*: the key consists of some *pair of instances* of objects or other concepts, and we measure some quality for each pair. For example, a distance matrix between cities has pairs of objects as a primary key and distance measurements as a foreign key. We call such relations *quantified relations*, and their qualities *binary qualities*.

**TABLE 1:** Concepts as types of relations of objects and measured qualities

	Unary	Binary
Measure	quality	quality
S (spatial geometry)	OS (object geometry)	OSO (path network)
B (Boolean quality)	OB (Boolean object quality)	OBO (Boolean network)
N (nominal quality)	ON (nominal object quality)	ONO (nominal network)
I (intensive quality)	OI (intensive object quality)	OIO (intensive network)
E (extensive quality)	OE (extensive object quality)	OEO (extensive network)

*Note*: Unary object qualities have a simple primary key, networks are binary object qualities (composite primary key).

**FIGURE 3:**
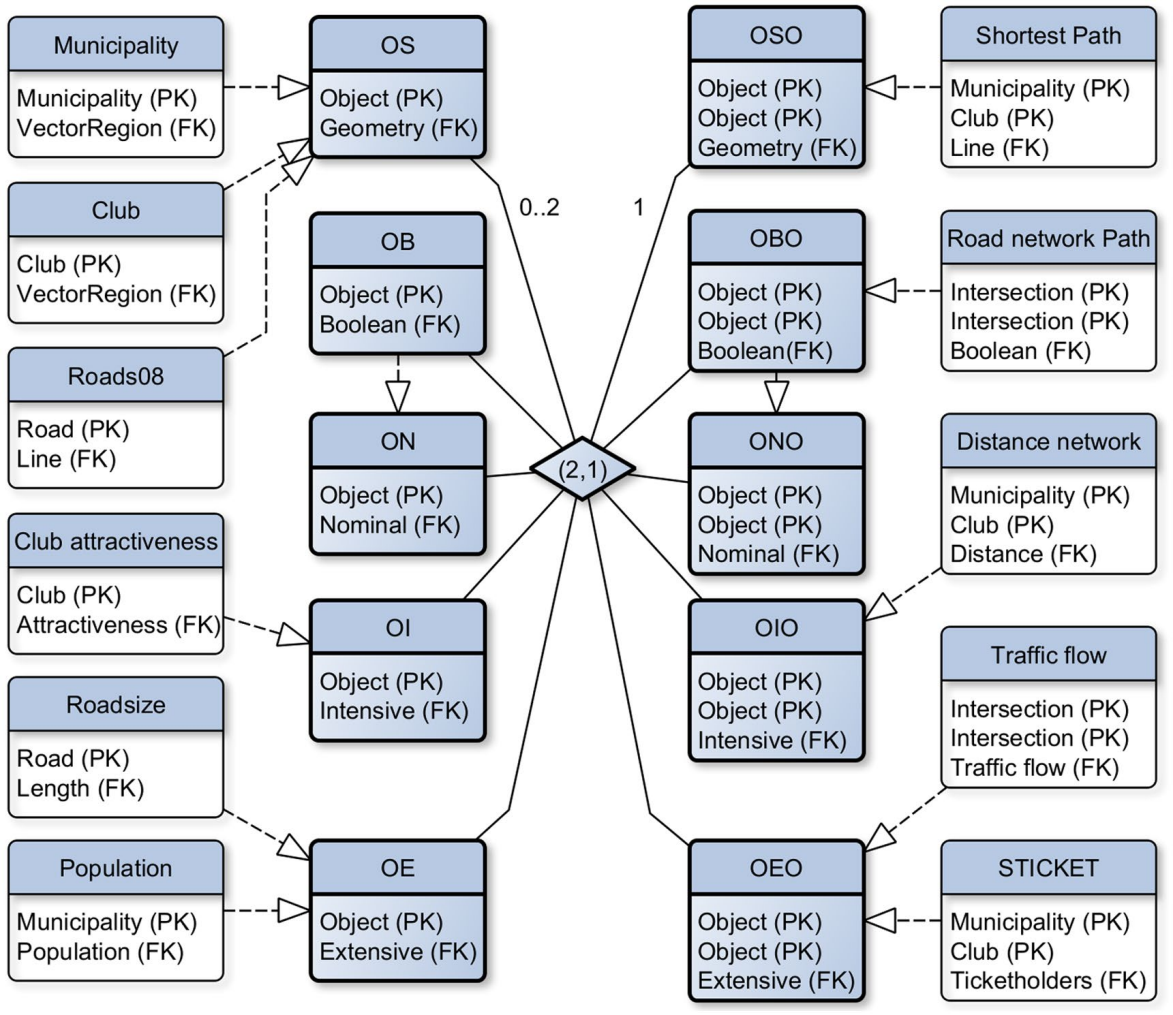
Entity relationship diagram of spatial network concepts, with realization examples (tables with primary/foreign keys) taken from our football scenario (see Section 6). Note how different tables can be realizations of a given concept

In principle, all core concepts can play a role in determining quantified relations. The measured quality, for example, can be generated by various kinds of concepts. To analyze a drainage network in a catchment area requires summation of a *hydrological field* (rainfall, water content) within the river catchment to determine network flow (Haggett & Chorley, 1969). To study movement or changes in a transport network, traffic or construction events need to be summarized. Furthermore, the primary key of a quantified relation can be formed by different concepts. Prominent GIS methods such as *visibility analysis* and *Euclidean distance* analysis can be conceived in terms of a Boolean or ratio scaled relation between locations in space. We might call the latter *relational fields*, given that they quantify a measure for pairs of locations, similar to ordinary fields quantifying a single location. Such hybrid models have been proposed earlier; see, for example, Cova and Goodchild ’s (2002) idea of *object fields*. However, within the limited scope of this article, we focus only on object‐based primary keys. This interpretation may correspond to a default understanding of spatial networks.

### 4.2 Measuring extensive and intensive network qualities

Unary and binary qualities can be measured on different levels, and in this way determine whether functions are applicable or not (Scheider & Tomko, 2016). For example, it is well known that different *levels of measurement*, including count, ratio, interval, ordinal and nominal, are relevant for understanding analysis in GIS (Chrisman, 2002). In this article we will make use of a *Boolean quality* including the values *true* and *false*, as well as *plain nominal qualities*, which correspond to qualities that are on a nominal level and not on any other level. We will also consider the regions of space that an object occupies as a measurable quality of that object.

The most important distinction for network qualities, however, is that between *spatially extensive and intensive* qualities (Scheider & Huisjes, 2019). Extensivity is known to influence the applicability of arithmetic functions, such as the possibility of forming sums:

- *Extensive qualities*, which are closely related to *amounts*, are ratio‐scaled qualities that are additive with respect to the *spatial extent* of non‐overlapping control units. An example of an extensive quality would be the population of administrative units. If we merge two such units into a larger one (assuming the units do not overlap), then their population counts sum in a corresponding way (Scheider & Huisjes, 2019). And the population count of a region shrunk to zero size becomes zero, making it ratio‐scaled (Chrisman, 2002). We consider extensivity as a class not only of unary qualities, but also of binary qualities or networks. Following this idea, *extensive binary qualities* are determined by the extents of the objects that constitute the network relation. Take the example of commuter flows: when merging a destination region (e.g.z a city) with a new destination (a satellite town), the commuter flow between origin and destination will increase by the sum of flows from the origin to the new destination.^5^
- *Intensive qualities*, in contrast, are ratio‐scaled qualities that do not sum when merging units. An example would be the percentage of elderly people of a municipality, or the distance to the closest sport club. When merging control units, the first quality needs to be aggregated using weighted averages, not sums. For spatial networks, we consider *intensive binary qualities*. An example would be the distance measured between two regions, which needs to be minimized, rather than summed, when merging one of these regions with others.

These ideas give rise to the relational types listed in Table 1. In Figure 3 these types are illustrated by entity relationship diagrams, with primary keys (PK) taken from data examples in our scenario (see Section 6). For example, layers of municipalities and football clubs are modeled as unary qualities with objects as primary key and some geometry as foreign key (OS). Road sizes and population numbers are examples of extensive unary qualities (OE). Distance networks (between municipalities and clubs), in contrast, correspond to intensive binary object qualities of type OIO, whereas traffic flows between road intersections correspond to extensive binary object qualities of type OEO. Binary qualities can also be Boolean, indicating whether paths go through a pair of objects, or consist of geometries that denote such a path (= path networks, type OSO).

**FIGURE 4:**
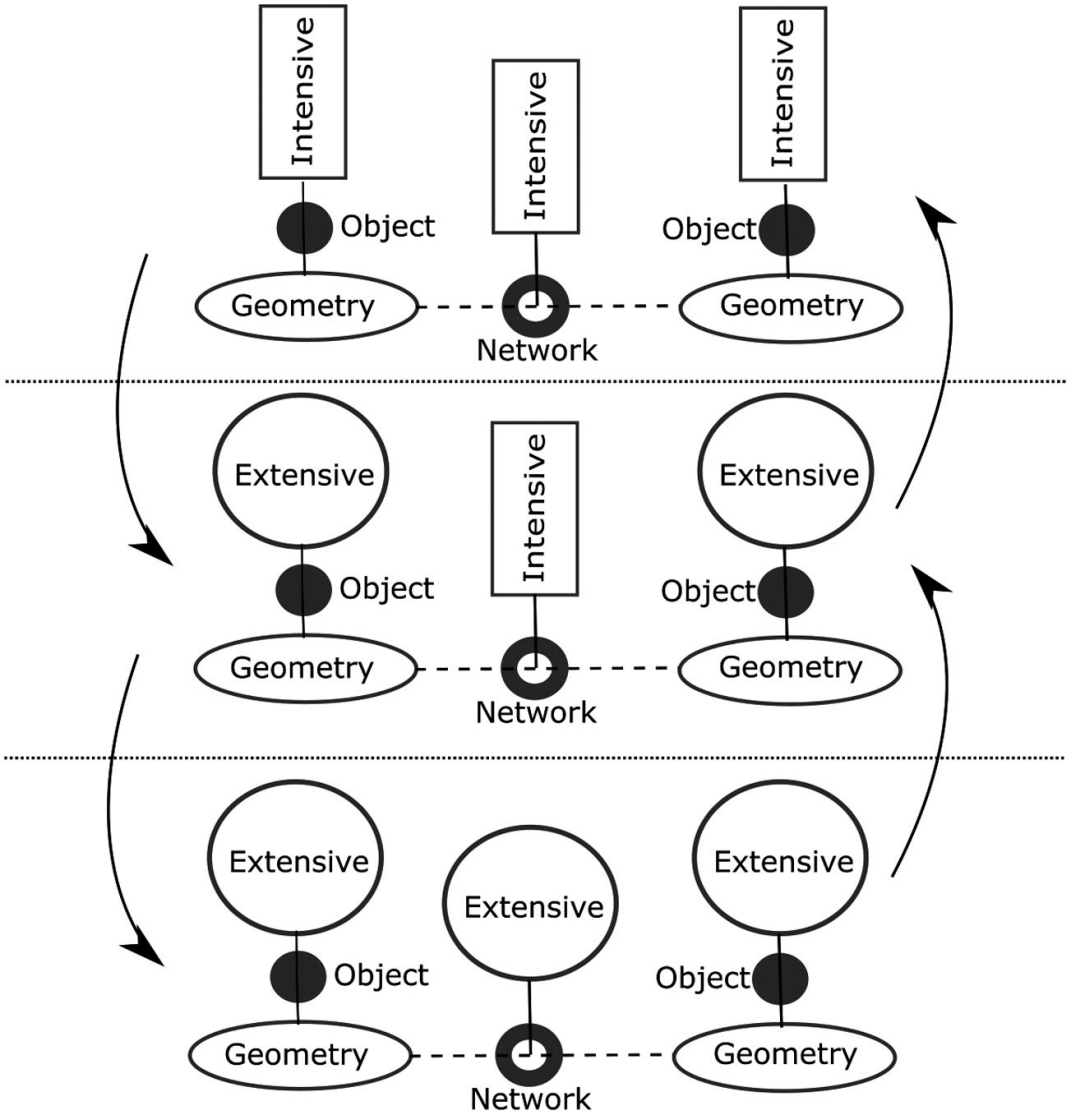
Modeling spatial networks in terms of qualities of objects and their relations. Both kinds of qualities can be extensive or intensive. Spatial network analysis essentially transforms these qualities into each other

Just as in relational algebra, we leave open how complete a given relation is with respect to its set of tuples and the domains that make up its key. Binary concepts that consist of an incomplete subset of the cross‐product of two given sets of objects are called *networks*. Networks might consist of only a single pair of objects as a key. Sometimes we want to be more exhaustive, and then the complete cross‐product of two sets of objects makes up the primary key of that relation, which we call a *matrix*. We use the star symbol * to refer to relations of that latter sort (e.g. OEO*).

Spatial network analysis, in essence, consists of *transformations* between such qualities (Figure 4). For example, a *catchment area analysis*, which computes network distances to the closest object in a layer, transforms an intensive (distance‐based) network between spatial objects with extensive quantities into intensive object qualities (distance to closest object). This corresponds to going from the middle layer to the upper layer in Figure 4. *Gravity models* (Batty, 1976), in contrast, allow us to estimate amounts of interactions between objects. In essence, they convert an intensive (distance‐based) quality between spatial objects with extensive quantities (middle layer) into some extensive quality (lower layer).

### 4.3 Representing object and network qualities as data types

The concepts discussed above are interpretations of input or output data of network functions, that is, they constitute *intermediary types*. Which formal type system should be used to add such interpretations to the data? A given core concept can be represented by various geometry types, and conversely, a given geometric model might be interpreted in terms of different concepts (Scheider et al., 2020). A field, for example, may be represented by vector lines or polygons (think about contours or land cover polygons), as well as by some raster layer. Similarly, networks may be represented by many kinds of geometries, not only by lines.^6^ And conversely, a line data set alone does not yet imply the existence of a network: to turn a roads file into a network, we first need to build a network topology. We take account of this representational variety simply by *three orthogonal semantic dimensions*: the core concept represented by a given attribute, its measurement level, and the geometry type of its layer. Each dimension forms an independent subsumption hierarchy, where subsumed classes are interpreted as sub‐classes. Classes can be combined arbitrarily between hierarchies, while leaf classes of one dimension are considered mutually exclusive.

**FIGURE 5:**
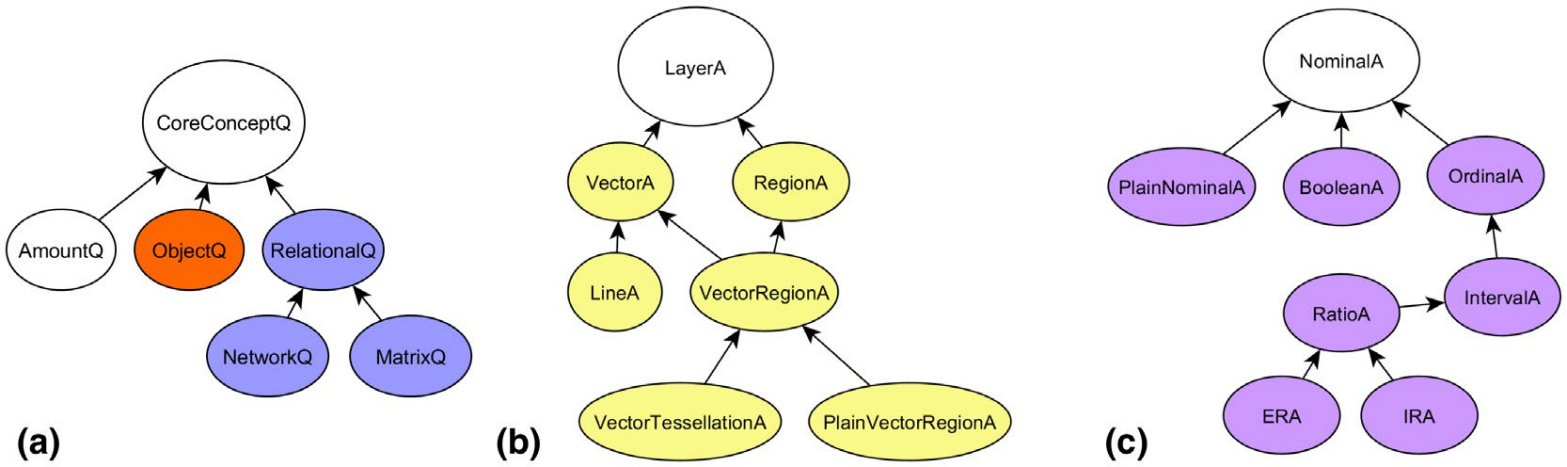
Three semantic dimensions of CCD types used in this article, including core concept, geometric types and measurement levels of attributes. Arrows denote subsumption relations. (a) The core concept represented by some geodata attribute. (b) The geometry type of some geodata attribute. (c) The measurement level of some geodata attribute

Dimensions were encoded by extending the core concept data types (CCD) ontology (http://geographicknowledge.de/vocab/CoreConceptData) (Scheider et al., 2020) with corresponding OWL classes (see Figure 5). The first dimension (Figure 5a) includes the hierarchy of core concept types. *CoreConceptQ* is the upper bound of this hierarchy and subsumes *ObjectQ* (object quality), *NetworkQ* (network quality) and *MatrixQ* (matrix quality). The latter two are subsumed by *RelationalQ* (≈ binary quality). *AmountQ* denotes amounts of objects or other content that is not bound to any object quality. We use this class to denote summary statistics. In the layer geometry dimension (Figure 5b), *LayerA* subsumes *LineA* (line attribute), *VectorTessellationA* (polygon tessellation attribute) and *PlainVectorRegionA* (attribute of a non‐tessellated polygon layer). The third dimension (Figure 5c) subsumes measurement levels of an attribute, with *NominalA* being the upper bound. *IRA/ERA* are considered subtypes of *RatioA* standing for intensive/extensive region attributes. *PlainNominalA* denotes nominal attributes that are not on a more specific measurement level. Conjunctions of these classes are used in the following to specify tasks, describe functions and compute workflows.

## 5 SPATIAL NETWORK TRANSFORMATIONS

Building on our model, we can distinguish available network functions based on how they transform one concept into another. This is done based on *type signatures* using the types from our model. The signatures of functions relevant to our scenario are given in Table 2, and each one is briefly explained below. The table contains software examples from ArcGIS as well as Flowmap.^7^ Tool annotations in RDF (http://www.w3.org/TR/rdf11‐primer/) are available online (https://figshare.com/s/1f794030aaf8b78175ab), including also geometry types for function inputs and outputs which are omitted here. Tasks illustrating their use are mentioned together with each function (see Appendix A).

**TABLE 2:** Functional signatures of basic spatial network transformations

Function	Software examples		Inputs			Outputs	
	ArcGIS	Flowmap					
Measure size	Calculate geometry attributes	Calculate length/size	Object regions OS			Object size OE	
Distance network	Build network	Import to flowmap	Object size OE	Object regions OS		Distance network OIO	
Network distance matrix	OD Cost matrix solver	Network distance matrix	Distance network OIO	Object regions OS		Distance matrix OIO*	
Functional clustering	–	Intramax analysis	Flow matrix OEO*	Object regions OS		Object nominal ON	
Catchment area	Closest facility analysis	Calculate catchment areas	Distance matrix OIO*	Object regions OS	Object regions OS	Object distances OI	
Network analysis	–	Transport network analysis	Distance network OIO	Object regions OS		Object distances OI	Boolean network OBO
Threshold amount (amount within distance)	(Service area analysis*)	Proximity count	Distance matrix OIO*	Object amounts OE		Object amounts OE	
Threshold distance (distance to amount)	–	Regular treshold distance	Distance matrix OIO*	Object amounts OE		Object distances OI	
Accessibility analysis	Summary statistics	Catchment profile	Object distances OI			Statistics I	
Flow matrix estimation (doubly constrained)		doubly constr. Gravity model	Distance matrix OIO*	Object amounts OE	Object amounts OE	Flow matrix OEO*	Attr./prod. scores OI
Flow matrix estimation (singly constrained)	Huff model	singly constr. Gravity model	Distance matrix OIO*	Object amounts OE	attr./prod. factors OI	Flow matrix OEO*	Object amounts OE
Flow summation	–	Interaction summation	Flow matrix OEO*			Object amounts OE	
Trip length analysis	–	Trip end ranking	Distance matrix OIO*	Flow matrix OEO*		Statistics I	
Trade area	(Probability based markets*)	Trade area analysis	Distance matrix OIO*	Flow matrix OEO*	Object regions OS	Object regions OS	
Flow assignment	–	Flow assignment to network	Flow matrix OEO*	Distance network OIO	Object regions OS	Flow network OEO	

I, intensive quality.

We start with basic functions that are underlying yet not usually considered to *be* network analysis. Usually, the first step in *constructing an intensive (distance) network* is to measure road lengths using street segment lines. We call this operation *measure size*, and it takes object regions (OS; in this case lines) and generates object sizes (OE; in this case lengths of lines), which are extensive measurements. Object sizes can then be used together with the geometry of their object regions in order to construct a distance network, based on topological (touch) relations between geometries. The latter are used to generate new (intersection) object pairs in the network, while the object sizes become *intensive distance qualities* of the network (OIO). This step corresponds to “building a topological network” in GIS. Following our logic of naming functions according to their outputs, we call it a *distance network* here. The *distance matrix function* takes an intensive network of distances (OIO), as well as a set of object regions (OS), and generates a matrix of network distances between all pairs of objects. Commonly this is the shortest path between these objects on the network, and involves, in case some objects are not in the network, also a metric distance measurement between these objects and their entry points to the network.

*Functional clustering* (Brown & Horton, 1970) between two locations in space is the reverse of the amount of interaction between them. For example, the *intramax method* developed by Brown and Masser clusters (adjacent) locations based on the amount (Masser & Brown, 1975) or relative amount (Masser & Brown, 1977) of interaction. It therefore takes an extensive (interaction) matrix, as well as some object regions, and generates a nominal object quality, where the nominal value indicates the cluster to which a given object belongs. Object regions are needed to determine whether objects are neighbors. An example is given with Task 6. A *catchment area* function takes an intensive (distance) matrix, some object regions as origins, as well as some object regions as destinations, and indicates, for each origin object, its distance to the closest destination object, as illustrated in Task 3. *Network analysis* does a similar thing, only based on an intensive distance network and some destination object regions, computing shortest distances to the closest object for all possible origins given within this network (Task 4). The resulting distance measurements on objects can be used to compute *accessibility statistics*. In addition, this function also outputs corresponding shortest paths given as a Boolean network, where *true* indicates that some path goes through the corresponding pair of objects. *Threshold distance* and *threshold amount* functions both take an intensive (distance) matrix and some extensive object quality (amount). The latter generates, for each object, the sum of amounts reachable within some distance, and the former the minimal distance to a given sum of object‐based amounts. In our scenario, an example is given in terms of fan potential analysis as part of answers to Tasks 1 and 2.

**FIGURE 6:**
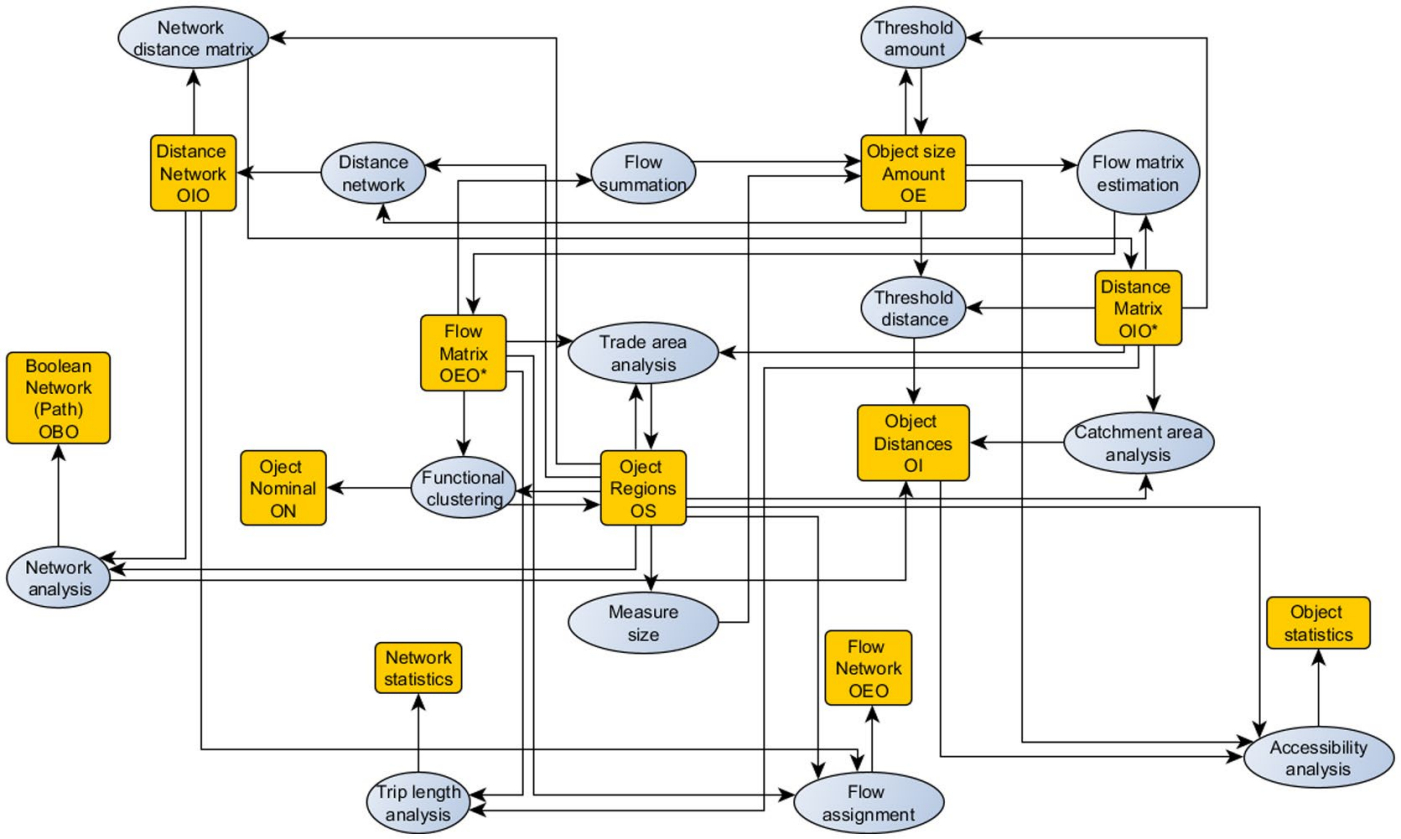
Computational diagram of spatial network transformations. Note that some signatures have been simplified in this diagram

A *doubly constrained flow matrix* function takes some intensive (distance) matrix and two extensive object qualities (amounts) and generates an extensive (interaction) matrix between these objects, as well as some attractiveness/productivity score on objects, which is intensive. For example, a gravity model (Batty, 1976; Huff, 1964; Wilson, 1974) can be used to estimate interactions between municipalities and football clubs based on both the number of ticket holders residing in each municipality and the number of tickets sold by each football club using some *distance decay function*. The parameter of the distance decay function is either given or fitted to a measured mean trip length. A *singly constrained* model, in contrast, takes some attractiveness/productivity score on destinations (origins) and some capacity on origins (destinations) to generate interaction estimations and amounts for destinations (origins). Examples are the different sorts of gravity models that can be used in Tasks 9, 10 and 11. *Flow summation* takes an extensive (interaction) matrix and sums up all outgoing flows to corresponding amounts on origin objects, as illustrated in Task 5. *Trip length analysis* is a statistical summary of the distribution of interactions over distances between objects, resulting in some trip statistics (average trip length (Task 7) or average trip end ranking), like the average car travel time for all trips being approximately 16 min. *Trade area* functions also take a distance and an interaction matrix as inputs, as well as some object regions, and determine some smallest (minimal distance based) object region that contains a particular sum of interactions. For example, it allows us to demarcate an area around each football club that contains a certain percentage of its closest ticket holders (Task 8). Finally, a *flow assignment* function takes some interaction matrix and some distance network as well as some object regions, and assigns flows to the network according to the shortest paths between flow origin and destination objects (Task 12). Functions are also summarized in the computational diagram in Figure 6.

Note that only three of the 15 functions in Table 2 require an actual transport network file. Most (10) of the other functions require a distance table that can be based on transport network distance but also on airline distances, time schedules, tariff structures or functional distances. This illustrates that spatial network analysis is much broader than implied by the common focus on transport networks. Furthermore, note that seven of the 15 functions did not have an equivalent in the standard software ArcGIS, though this functionality can of course be reprogrammed.

## 6 SPECIFICATION OF WORKFLOW TASKS IN FOOTBALL SCENARIO

Starting from a simple data source, we went through 12 different analytical tasks^8^ as an empirical basis for evaluating our model. We begin with a description of the available data sources. Note that an in‐depth study of the data and the results is beyond the scope of this article.^9^

### 6.1 Data source specification

There are five different data sources, which were interpreted in terms of the following types in our model:

- *MUNCLUB* (ObjectQ,VectorTessellationA,PlainNominalA). Polygon layer containing 489 municipalities, plus the four‐digit postcode areas of 37 professional football clubs in the Netherlands. The “LABEL” field contains the residential municipal name or football club name, and the “FC” field is 1 in the latter and 2 in the former case. Conceptually, this corresponds to a collection of objects, including *ON* and *OS*. Municipalities form a vector tessellation of the area.
- POP_2003∗MUNCLUB (ObjectQ,VectorTessellationA,ERA). Table POP_2003 contains the total population of each municipality (CBS Statline), corresponding to an extensive object‐based vector tessellation attribute, and corresponding to *OE*.
- ROADS08 (ObjectQ,LineA,PlainNominalA). The Dutch road transport network ROADS08 (BASNET by Adviesdienst Verkeer en Vervoer) contains names of roads and line geometries, thus including both *ON* and *OS*. Note that roads are conceived as linear objects, not networks.
- STICKET2 (MatrixQ,VectorTessellationA,ERA). This spatial interaction table of the Royal Dutch Football Association contains the total number of season tickets for each combination of residential municipality and club. In total, in 2003, there were 349,538 ticket holders distributed over 3,459 different combinations of municipality and club. The interaction table reports numbers of ticket holders. Thus it corresponds to an extensive matrix quality OEO*.
- NEWCLUBS (ObjectQ,VectorTessellationA,IRA). This gives the hypothetical attractiveness score for each football club (*OI*) in a scenario where the lower professional league is abolished.

Though these sources cover only a limited set of types, further types of data are generated as part of the workflows described below.

### 6.2 Specification of analytical tasks and expert workflows

Each task was described by a unique question (workflow task; see Table 3 and Appendix A). The latter was then *specified* in terms of our type model (CCD), including *input data types*, *goal types* and (optionally) requests for intermediate data types that should be *used* in the workflow. Specifications were later used as a basis for automatic workflow synthesis. Furthermore, we manually generated one expert workflow for each question (examples below). In Appendix A we explain in more detail how each task specification reflects the information given in the question, which computational steps are needed to answer it, and how the resulting maps look.

#### 6.2.1 Distance‐based analysis

We first considered analytical Tasks 1–4 that exploit distances between residential areas and football clubs measured on a road network, in addition to amounts measured at origins or destinations. Workflow tasks include the assessment of fan potentials and accessibility analysis. Computationally, these tasks require the generation of a *distance matrix* between objects, by computing shortest path distances on the road network and including the last mile between road intersections and these objects (*use types*). To assess *fan potentials*, the goal types are extensive/intensive object qualities. *Accessibility analysis* requires intensive (distance‐based) object qualities, represented either as regions (municipality level) or lines (street level). Workflows for Tasks 1–3 were discussed in Section 3.

**TABLE 3:** Spatial network analysis tasks for synthesizing workflows

	Task		
Task category	subcategory	Workflow task	Task specification
Distance‐ based analysis	Fan potential	1 "What is the potential number of fans within a travel distance for each municipality?"	input: (1) ROADS08, (2) POP_2003
			goal types: OE (ObjectQ, RegionA, ERA)
			use type: OIO* (MatrixQ, IRA)
		2 "What is the potential minimal travel distance to reach a certain number of fans for each municipality?"	input: (1) ROADS08, (2) POP_2003
			goal types: OI (ObjectQ, RegionA, IRA)
			use types: OIO* (MatrixQ, RegionA, IRA)
	Accessibility	3 "What is the accessibility of clubs from each municipality?"	input: (1) ROADS08, (2) MUNCLUB
			goal types: OI (ObjectQ, RegionA, IRA)
			use types: OIO* (MatrixQ, RegionA, IRA)
		4 "What is the accessibility of clubs from each road?"	input: (1) ROADS08, (2) MUNCLUB
			goal types: OI (ObjectQ, LineA, IRA)
			use types: OIO (NetworkQ, LineA, IRA)
Interaction‐ based analysis	Flow summation	5 "What is the number fans for each municipality?"	input: (1) STICKET2
			goal types: OE (ObjectQ, RegionA, ERA)
	Functional clustering	6 "To which functional cluster does a club belong?"	input: (1) STICKET2, (2) MUNCLUB
			goal types: OS, ON (ObjectQ, RegionA, PlainNominalA)
	Trip length distribution	7 "What is the average travel time to/trip rank of a club?"	input: (1) ROADS08, (2) MUNCLUB, (3) STICKET2
			goal types: I (IRA)
	Trade area analysis	8 "What is the area enclosing 60% of the number of fans closest to each club?"	input: (1) MUNCLUB, (2) ROADS08, (3) STICKET2
			goal types: OS, ON (ObjectQ, RegionA, PlainNominalA)
			use types: OEO* (MatrixQ, ERA), OIO* (MatrixQ, IRA)
Flow generation	Gravity modeling	9 "What is the potential number of fans in each municipality for each club assuming distance decay?"	input: (1) MUNCLUB, (2) ROADS08, (3) STICKET2
			goal types: OEO* (MatrixQ, RegionA, ERA)

		10 "What is the attractiveness of clubs for fans?"	input: (1) MUNCLUB, (2) ROADS08, (3) STICKET2
			goal types: OI (ObjectQ, RegionA, IRA)
		11 "What is the potential number of fans for each club when the lower professional league is closed?"	input: (1) MUNCLUB, (2) ROADS08, (3) STICKET2,
			(4) NEWCLUBS
			goal types: OE (ObjectQ, RegionA, ERA)
			input: (1) MUNCLUB, (2) ROADS08, (3) STICKET2
		11a (a more challenging version of 11)	goal types: OE (ObjectQ, RegionA, ERA)
			use types: OI (ObjectQ, RegionA, IRA)
	Traffic load analysis	12 "What is the potential traffic load for each road assuming fans travel by car at the same time?"	input: (1) MUNCLUB, (2) STICKET2, (3) ROADS08
			goal types: OEO (NetworkQ, LineA, ERA)


*Note*: Tasks were formulated as questions and specified using CCD types.

#### 6.2.2 Interaction‐based analysis

**FIGURE 7:**
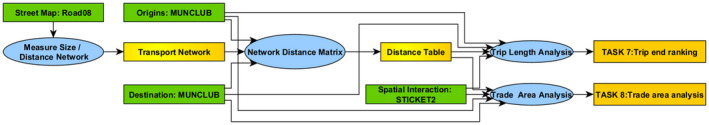
Expert workflows solving Tasks 7 and 8

Here we focus on tasks that analyze spatial interaction or flows between residential areas and clubs, in addition to the network distance, making use of a (measured or modeled) *interaction matrix* (type OEO*). This includes *flow summation* (Task 5) to summarize flows of destination/origin amount totals, and which was specified by requesting extensive object qualities as goal type. *Functional distance clustering* (Task 6) was specified by requesting nominal values (cluster identifiers) for objects. *Trip length distribution* (Task 7) was specified by requesting some intensive measure. Finally, *trade area analysis* (Task 8) was specified by requesting an object‐based region. Workflow solutions for Tasks 7 and 8 are shown in Figure 7.

#### 6.2.3 Flow generation

**FIGURE 8:**
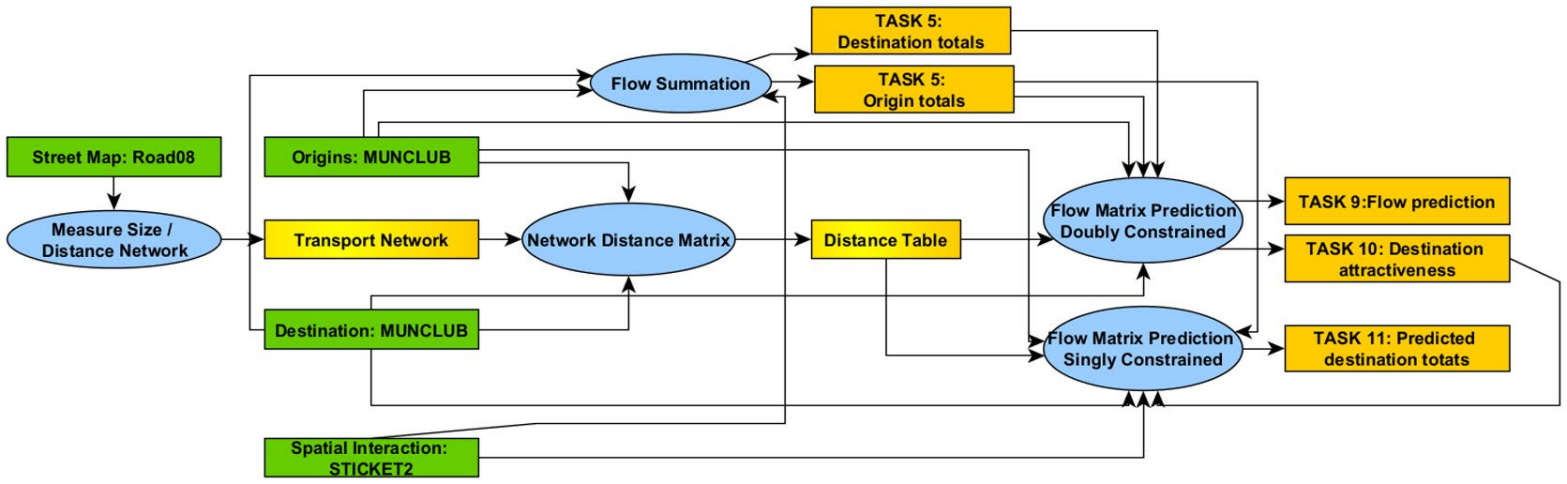
Expert workflows solving Tasks 9–11

The final type of analysis provides ways of estimating interactions from other kinds of spatial information. Expert workflows solving these tasks are depicted in Figure 8.

The task of estimating the potential number of season ticket holders (Task 9) was specified by requesting an extensive matrix. Another task was to estimate relative *attractiveness scores* (Task 10) for clubs, based on the product of (club or municipal) amount and their matching “balancing” factor. This was specified by an intensive object quality. Finally, suppose the lower professional football league is abolished and their attractiveness becomes zero. What will happen to the fans and the remainder of the clubs? To answer this question, the task (Task 11) was to generate an extensive object quality (goal).*A more challenging version of the same task* (Task 11a) is to start without manually generated attractiveness scores, but require the generation of attractiveness scores in an intermediate step, via *use types*. The final flow generation task takes an interaction matrix between municipalities and clubs, as well as a street network as input, and generates finer‐grained flows between road intersections, based on assuming that trips are made on the shortest paths on this network. This task is called *traffic load analysis* (Task 12), specified by requesting an extensive network quality on lines.

## 7 EVALUATION

**FIGURE 9:**
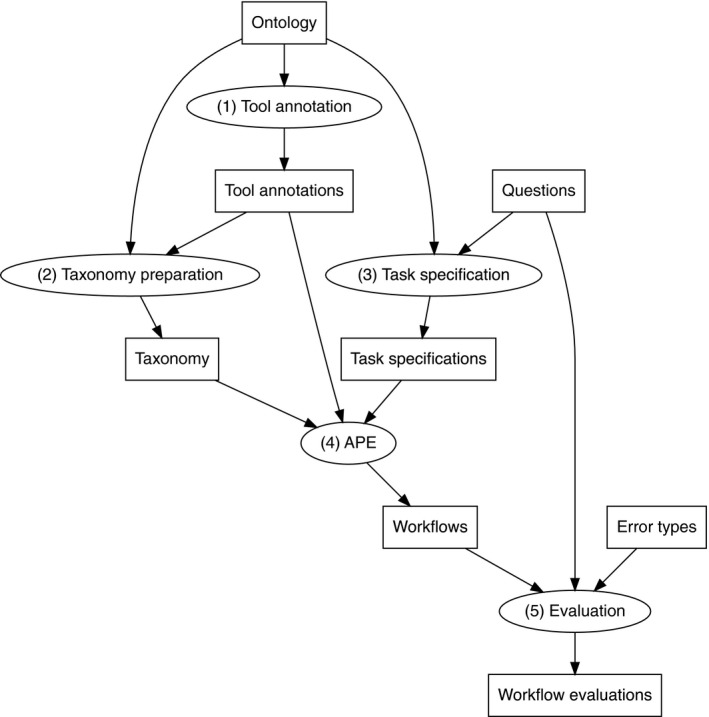
A summary of our ontology evaluation framework for workflow synthesis. For an ontology, five steps are performed. All steps are performed both for the ontology and the benchmarks to measure improvements (Kruiger et al., 2021)

When thinking is turned into workflows, concepts need to be translated into concrete tools and data sources. Our hypothesis is that common geodata models alone, as well as graph‐theoretic models, *are insufficient* to perform such a translation. To test this hypothesis, we follow an approach of workflow synthesis quality assessment that was developed in Kruiger et al. (2021). An overview of the evaluation process is shown in Figure 9. We compare the quality of automatically synthesized workflows that were generated using our conceptual model against two benchmark models. In this section, we explain the synthesis algorithm, the benchmark models and our workflow quality assessment approach.

### 7.1 Synthesis algorithm and workflow repository

We used a workflow composition algorithm as described in Kasalica and Lamprecht (2020a). Automated Pipeline Explorer (APE, https://github.com/sanctuuary/APE) generates sequences of tool applications satisfying logical (type) constraints as used in our task specification (input types, output types, use types). The latter are expressed in semantic linear‐time logic using the classes of our ontology. The three semantic dimensions of the CCD model were used independently as constraints for this kind of reasoning, and class combinations were automatically interpreted as class conjunctions. Furthermore, leaf classes in one dimension were interpreted as mutually exclusive and jointly exhaustive. In APE, workflow models satisfying these task specifications are generated with increasing size, drawing from a repository of tool signatures (see Table 2) annotated with the same types. The maximum number and size of workflows were given as parameters. In our test, we generated five workflows up to a length of 10 tool applications for each variant of a task. More workflows increased only the amount of soft errors (see Section 7.3). Furthermore, we used the constraints that all given input data should be used in the workflow, and that at least one of the data instances that are generated as output, per tool, has to be used. The workflow synthesis repository with all resources is available online (https://figshare.com/s/aea5c00a9858db69e37f), including task specification files (*ape.configuration and constraints.json*) for the 12 tasks as well as resulting workflows, for both CCD and the benchmark solutions. Workflow outputs are generally encoded as directed acyclic graphs with function applications as vertices. Examples of automatically synthesized workflows are shown in Appendix B. In APE, workflows can also be exported in a serialized form, as an executable script. This requires, however, a way to deal with function parameters (see the discussion below).

### 7.2 Benchmarking

We compared the synthesized workflows from our model against workflows obtained under the exact same conditions, except that we used some modified type system reflecting the kind of information available in current data models used to represent spatial networks. We considered two benchmark variants:

1. Geometric benchmark (abbreviated *bench*). This is a proper subset of CCD where the two conceptual dimensions (including core concepts and measurement levels) were removed, including only one dimension related to geometry types, namely the distinction between raster and vector attributes, as well as between point, line and region attributes (see Figure 5b). The distinction between *VectorTessellationA* and *PlainVectorRegionA* was also removed, since it does not occur in current data structures.
2. Embedded graph benchmark (abbreviated *graph*). This version retains the idea of a graph embedded into geometric space. We distinguish between nodes and directed edges (≈ relations between nodes) based on the core concept superclasses *ObjectQ* and *RelationalQ* (see Figure 5a) respectively, together forming one dimension. Furthermore, nodes as well as edges can be embedded by either of the geometric types in the geometric benchmark. This is encoded by taking the geometric benchmark types as a second dimension.

Using these benchmark versions of the ontology, we manually created corresponding tool annotations by substituting every type with the least upper bound (supremum) concept that is still in the corresponding benchmark ontology. In the same way, we generated benchmark versions of all task specifications, by substituting input, use and goal types with their benchmark equivalents, respectively.

### 7.3 Evaluation metrics and quality assessment

We treated workflow synthesis like a retrieval process, measuring its quality with respect to an expert judgment and considering expert workflows produced independently with Flowmap. We decided to measure both precision (the proportion of retrieved answers that are correct given all retrieved answers) as well as recall (the proportion of retrieved answers that are correct given all correct answers).

To assess recall, an expert on spatial network analysis went through the tasks ahead of our study and manually generated a gold standard of expert workflows, using the set of spatial network functions in Table 2. Afterwards, when going through the synthesized workflows for each task, the expert simply indicated whether one of them corresponded to the expert workflow for this task.

To assess precision, our expert assessed synthesized workflows individually based on different error types. We used three error types on two different severity levels, which are summarized and illustrated in Table 4.

**TABLE 4:** An overview of the different error types (figures in Appendix B)

**Error severity**	**Error type**	**Example workflows**
Hard	Syntax	Figure B3
	Semantic	Figure B2
Soft	Redundancy	Figure B4

*Hard errors* are critical errors which result either in a wrong or non‐meaningful answer, or in a workflow that is non‐executable due to wrong data formats. We distinguish two kinds of hard errors: *syntax* errors, which have a part of the workflow that cannot be executed because a tool is incorrectly applied, and *semantic* errors, which produce a meaningless or invalid answer for the given question. *Soft errors* are non‐critical errors where workflows *do* entail a correct answer, but which are in some sense of lesser quality. We focused on *redundancy* errors, where workflows make use of unnecessary tool applications.

## 8 RESULTS

Evaluation result data sets are available online (https://figshare.com/s/7f44d57de058b51c19e3). In Table 5, evaluation results for each task variant are shown as a statistic over the first five workflows generated using each task specification. *Num* indicates the number of workflows for each task variant, which can be less than five in case not more options were found. *Semantic error* denotes the number of semantic errors in these workflows, *Syntactic error* denotes the number of syntactic errors in these workflows, *Correct* denotes the number of workflows without hard errors, *Rdn* denotes the number of correct workflows with redundancy errors. *Expert solution* denotes the number of correct workflows that correspond to an expert solution. *Expert order* denotes the order of occurrence of the first expert solution, in case it occurred within the set of generated workflows, and ∞ otherwise.^10^ Results are listed for workflows generated with the CCD model (CCD[*x*]), the embedded graph model (graph[*x*]), and the geometric benchmark model (geom[*x*]). In the *total* row we summed up all workflow counts and averaged the length and order measurements for each of these three test variants. In total, we checked the quality of *181 workflows*. Our interpretation of these results is summarized as follows:

- Our study shows that the CCD model is capable of *reproducing at least one expert workflow for each single task* (see the *expert solution* column). In total, 22 expert workflows could be recalled by the CCD model. Removing duplicates, this amounts to 13 unique expert workflows (including Tasks 11 and 11a; see Figure 14), which is a recall of 100%. This is in stark contrast to the geometric benchmark, which only produced a single expert solution (for Task 5) over all 12 tasks (recall 8%), as well as the embedded graph model, which found four expert solutions (recall 33%). Whether the exceptionally high recall value of the CCD model can be sustained for larger sets of expert workflows or other kinds of tasks remains to be seen. However, it shows that our model indeed is capable of accounting for a significant amount of such expert knowledge.
- Furthermore, the expert solutions that were found by CCD *appear very early in the process* (see the *expert order* column). Most often they appeared as the first solution, except for Tasks 10 and 11a, where they appeared as number 5 and 2 in the row. In the four cases in which the graph model was able to produce experts solutions, these were generated in places 5, 2, 3, 5. This indicates that despite of the presence of semantically incorrect or redundant workflows, high‐quality solutions produced by the CCD model may be filtered out simply by constraining the number of workflows generated.
- CCD solutions are on average much longer than benchmark workflows (4.5 nodes compared to 1.8 in the geometric and 2.5 in the graph model) (see the *Avg length* column). This indicates that the CCD ontology adds more constraints to the space of workflow composition, and thus contains more information than both the geometric and the graph model.
- Thirty‐six out of 61 CCD workflows (59%) were correct solutions of the task (without any semantic or syntactic errors) (see the *Correct* column). This is again in stark contrast to the geometric model, with a precision of less than 2%, and also to the graph model, with a precision of 6%. This indicates that without deeper semantics, it becomes nearly impossible to generate high‐quality solutions, even if using an embedded graph. Furthermore, since errors tend to occur with larger solutions, the precision of the CCD model dramatically increases to 84% (11 out of 13) when selecting the first workflow as a solution for each task. Still, there remain quite a lot of semantic and syntactic errors in the CCD solutions. The 13 semantic errors were due to missing workflow constraints implicitly contained in the task (see the discussion below). The 16 syntax errors were mainly due to the fact that some of the computational functions in our model, which are treated independently, are actually not implemented in terms of independent components in the Flowmap software.^11^ In consequence, some possible combinations and repetitions of these tools in our model are actually syntactically impossible in Flowmap. These errors can be easily avoided by forcing the tools to be used only once or only in conjunction with others. Furthermore, syntactic errors due to repetitions can be considered redundancy errors. If we count these errors as redundancy errors instead, the hard error rate of the CCD solutions falls by 10, resulting in a precision of ≈ 75% (46 out of 61).
- *Redundancy errors* occur within CCD workflows mainly because CCD imposes increased constraints on the workflow composition process, and so the only possibility of generating longer workflows is to repeat function applications. This is compatible with earlier results (Kruiger et al., 2021). The problem can be handled by further restricting the number of workflows produced for each task.

Regarding the validity of these results, we would like to add the following considerations. First, one may ask whether the chosen benchmark for comparison is of sufficient quality. Our argument is that the benchmarks cover precisely the concepts used and available in current spatial network information systems. These are, on the one hand, geometric data types, and on the other hand, graph‐theoretic models. We were rather lenient with the combinability of graph elements and geometry types to distinguish functions, which in practice is rather more restricted. Second, one might ask whether our chosen tasks and scenarios are not too limited in range. Our list indeed lacks some common network functions, including more complex routing functions, such as traveling salesman or Chinese postman routing, or location allocation methods. However, the first two of these can be seen as a special case of the network distance matrix function. Shortest‐path routing deals with a single origin and destination and some path network (OSO) as output that contains all trips as geometries between origin and destination objects. In the traveling salesman variant, the only thing added is another object input, namely, the objects to be visited on a tour. Location allocation functions are methods to place objects in respect of both amounts and distances, and thus should also fit well into our framework. Third, regarding the complexity of our tasks, we believe they correspond to the level required in practice. Nevertheless, it should be investigated in the future how longer tasks and larger repositories of functions influence the quality of workflows. And fourth, in the practice of spatial network analysis, parameter settings and fitting of parameter values (e.g. the distance decay parameter for gravity models) and manual interventions are essential parts of a workflow. In this respect, our model still commits to a considerable simplification, leaving completely automatized workflow synthesis beyond current reach. However, this could be addressed in the future by incorporating abstract parameter semantics. What kinds of concepts could be used for this purpose, however, is an open question. Finally, in compliance with previous results (Kruiger et al., 2021), it seems that the amount of semantic errors can only be further reduced when incorporating information about the type of transformation. As shown in Figure 15, this workflow for Task 10 fails because the threshold distance function has the same result type as the (required) attractiveness score of the doubly constrained flow matrix function. To prevent this error, we would need to distinguish between measuring threshold distances and measuring attractiveness, which is beyond the current model. However, the workflow synthesis algorithm would allow such tool constraints to be incorporated (Lamprecht et al., 2010).

**TABLE 5:** Results of evaluating the core concept (CCD) model of spatial networks against the benchmark models

Task	Variant	Num	Avg length	Semantic error	Syntax error	Correct	Rdn	Expert solution	Expert order
1	CCD1	5	4.8	2	1	2	1	1	1
	graph1	5	3.0	4	5	0	0	0	∞
	geom1	5	1.8	5	5	0	0	0	∞
2	CCD2	5	4.6	3	2	1	0	1	1
	graph2	5	3.0	5	5	0	0	0	∞
	geom2	5	1.8	5	5	0	0	0	∞
3	CCD3	5	5.6	2	3	2	1	1	1
	graph3	5	3.0	4	5	0	0	0	∞
	geom3	5	1.8	5	5	0	0	0	∞
4	CCD4	5	4.6	0	3	2	1	1	1
	graph4	5	2.8	0	4	1	0	1	5
	geom4	5	1.6	4	5	0	0	0	∞
5	CCD5	1	1.0	0	0	1	0	1	1
	graph5	5	1.6	2	4	1	0	1	2
	geom5	5	1.0	4	3	1	0	1	1
6	CCD6	5	3.0	0	4	1	0	1	1
	graph6	5	1.0	4	4	1	0	1	3
	geom6	5	1.0	4	5	0	0	0	∞
7	CCD7	5	4.8	1	1	4	3	1	1
	graph7	5	2.4	4	5	0	0	0	∞
	geom7	5	2.8	0	5	0	0	0	∞
8	CCD8	5	4.8	0	0	5	4	1	1
	graph8	5	2.4	4	5	0	0	0	∞
	geom8	5	2.0	4	5	0	0	0	∞
9	CCD9	5	5.2	0	1	4	0	4	1
	graph9	5	2.6	5	5	0	0	0	∞
	geom9	5	2.0	5	5	0	0	0	∞
10	CCD10	5	5.0	4	0	1	0	1	5
	graph10	5	2.4	5	5	0	0	0	∞
	geom10	5	2.0	5	5	0	0	0	∞
11	CCD11	5	5.2	0	0	5	1	4	1
	CCD11a	5	6.0	1	0	4	0	4	2
	graph11	5	3.0	5	5	0	0	0	∞
	geom11	5	2.0	5	5	0	0	0	∞
12	CCD12	5	3.8	0	1	4	3	1	1
	graph12	5	2.8	0	4	1	0	1	5
	geom12	5	1.8	3	5	0	0	0	∞
Total	CCD	61	4.5	13	16	36	14	22	1.4
	graph	60	2.5	42	56	4	0	4	‐
	geom	60	1.8	49	58	1	0	1	‐

*Notes*: Each task variant included the first five workflows generated by APE under the given specification. In total, 181 workflows were evaluated. See text for explanation.

## 9 DISCUSSION AND CONCLUSION

In this article we suggested and tested the idea that spatial network analysis, as implemented in GIS, and as envisioned by early writers in network‐related geography, can be fruitfully understood as a repertoire of functions that transform between relations of objects and their qualities. Qualities can be unary or binary, extensive or intensive (depending on whether they are additive with respect to the spatial extent of the controlling objects), and on different levels of measurement. To this end, we extended the core concept data types ontology with new classes along three semantic dimensions, including core concept, measurement level and geometry type. We also included two benchmark models, one of them corresponding to a geometrically embedded graph.

We tested our model against the benchmarks on a scenario with 12 different network analysis tasks. We evaluated automatically synthesized workflows by expert judgements and by comparing them with independently generated expert workflows. Despite its simplicity, we demonstrated that the model helps us not only to more clearly understand the underlying functions, but also to automate spatial network analysis to a degree that can support analysts in answering questions. Our model distinguishes (question 1) 12 network analysis tasks in terms of input/output and intermediary types, which was sufficient to instruct corresponding workflow synthesis. Only in few cases (e.g. Task 10) was the model not able to distinguish between tasks that should result in different workflows. Furthermore, the model was sufficient (question 2) to distinguish between all relevant spatial network functions, except for functional differences that depend on *function parameters* or *type‐equivalent transformations*, (e.g. threshold distances and attractiveness scores) which were not distinguished in this study. Furthermore, regarding the quality of synthesized workflows (question 3), results show not only that the model was capable of regenerating all expert workflows, but also that the semantic depth added by our model over and above graph theory is crucial for high‐quality workflows, improving their accuracy from 6% to 60%, and potentially over 75% under certain adjustments.

To enable fully automatized workflows and executable workflow scripts, there are still several open issues. First, future work should focus on models for incorporating method parameters (which were not considered here) and for removing remaining syntax errors. To remove the considerable amount of semantic errors, the model needs to be extended to types of network transformations. Modeling parameter semantics is closely related to a transformation model, because function parameters are often functions themselves (e.g. “averaging” trip lengths versus “taking the median” of trip ranks). We are currently working on a transformation algebra that is based on a higher‐order type system for specifying such conceptual transformations. Finally, tool annotations should be extended to encompass further relevant software for spatial network analysis, including QGIS, ArcGIS and Python libraries, allowing for cross‐software comparisons.

What are the wider implications of these results? We see our work in the context of symbolic AI for GIS (Janowicz et al., 2019). For purposes of GIS automation, we can learn from this study that the know‐how required to deal with spatial information generally goes beyond knowing the computational procedures or having the data. Thus reducing know‐how to knowledge extraction runs the risk of underestimating this task. This is especially important in an age where intelligence tends to be reduced to a variant of machine learning. By reducing analysis to the computational process on data, we disregard the underlying reasoning process that is necessary to arrive at meaningful results. As our study demonstrates, this reasoning process requires concepts *instilled into* data, *not extracted from* data. Correspondingly, while Janowicz et al. (2019) claim that “GeoAI research will have to make a case for spatially explicit models,” our study clearly shows that for purposes of automation, explicit spatial models are beyond question, and that even such models can still be insufficient. While we have made a suggestion for the kind of knowledge lacking, it remains unknown what we will lose once our network experts are substituted by machines.

## Figures and Tables

**FIGURE A1:**
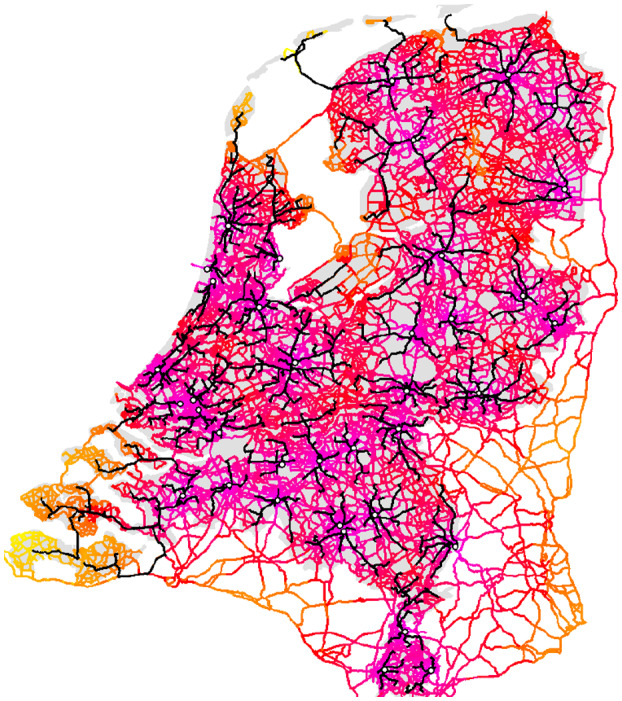
The map shows for each road segment the travel time to the closest football club, from more than half an hour in yellow, via half an hour in red, to less than 5 min in purple. The black lines indicate the shortest path from each municipality to its closest football club

**FIGURE A2:**
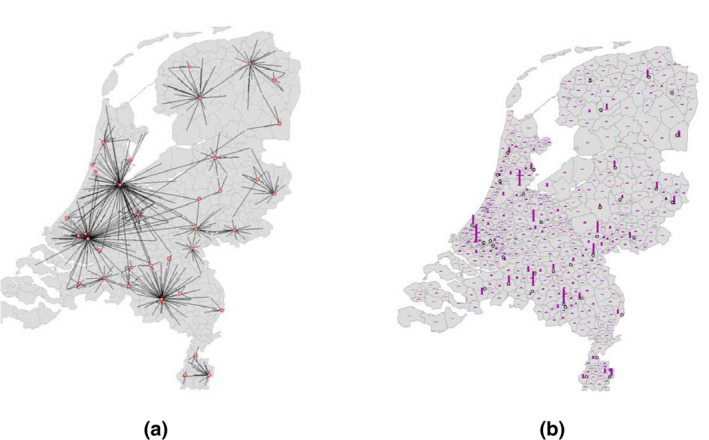
Numbers and flows of football fans in the Netherlands: (a) Flow of ticket holders, displayed as desire lines; and (b) Numbers of ticket holders by municipality, displayed as a bar chart.

**FIGURE A3:**
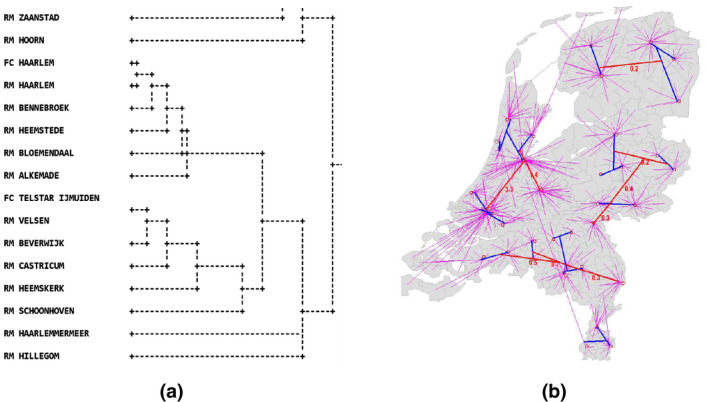
Functional distance‐based clustering. (a) Functional distance clusters. (b) Functional distance clusters on map

**FIGURE A4:**
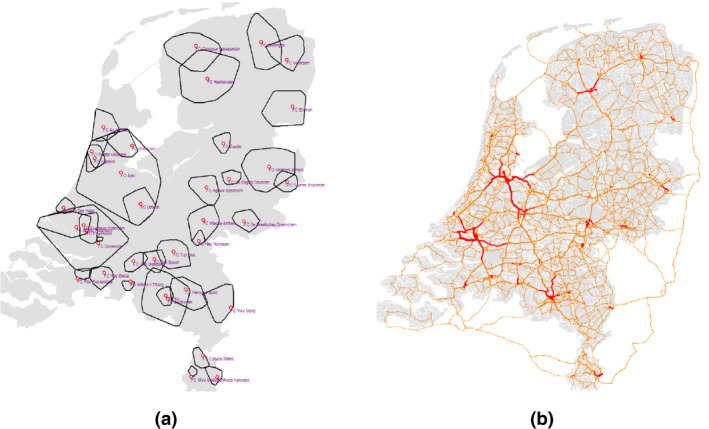
Trade areas of football clubs and flow assignments of football fan trips in the Netherlands. (a) Trade areas of football clubs: convex hulls around origin municipalities that demarcate the closest 60% of interactions with destinations. (b) Flows of football ticket holders assigned to street network. Amounts designated by line width and color hue (red, 3,000–49,000 ticket holders)

**FIGURE B1:**
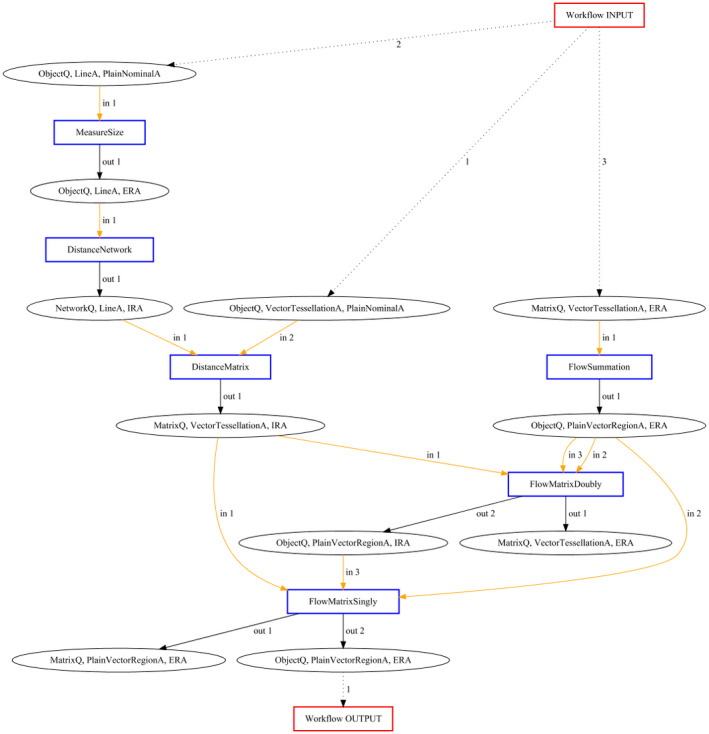
Example of an *expert solution* for Task 11a automatically generated with the CCD model in APE. Here we model potential numbers of ticket holders for each football club in a scenario where some clubs are closing. We start from a roads file (2), municipalities and clubs given as an object tessellation (1), and a ticket interaction table between these objects (3). Attractiveness scores for clubs (used to obtain amounts in the last step applying a singly constrained gravity model) are generated on the fly using a doubly constrained gravity model

**FIGURE B2:**
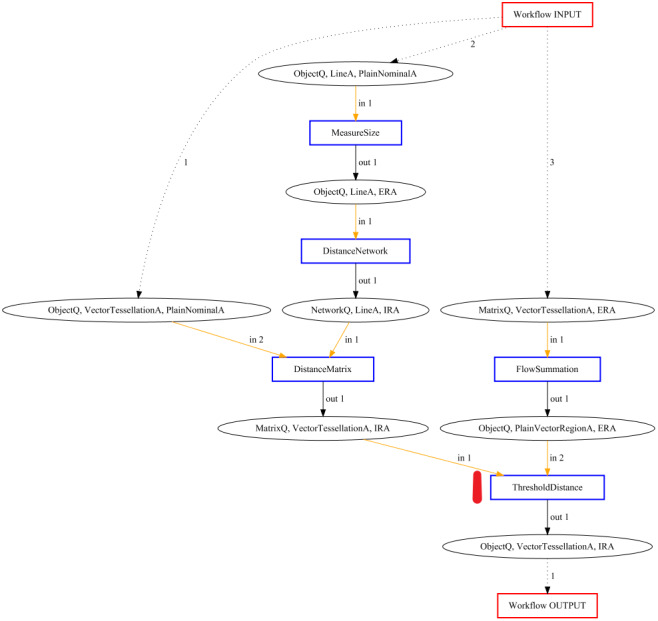
Example of a *semantic error* produced by the CCD model for Task 10. The task is to generate attractiveness scores for clubs, based on a municipality/club tessellation (1), a roads file (2) and am interaction table (3). The problem is that threshold distances are not attractiveness scores, and that the task specification lacks semantic detail to prevent this confusion

**FIGURE B3:**
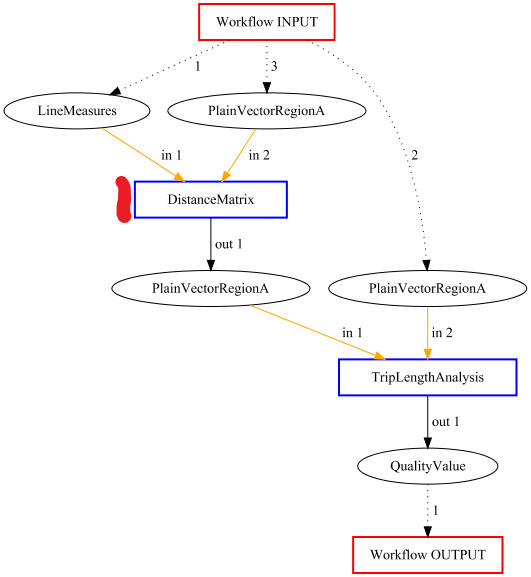
Example of a *syntax error* produced by the geometric benchmark model for Task 7. The distance matrix function needs a topological network as data input, but it is given a roads file, resulting in a syntax error

**FIGURE B4:**
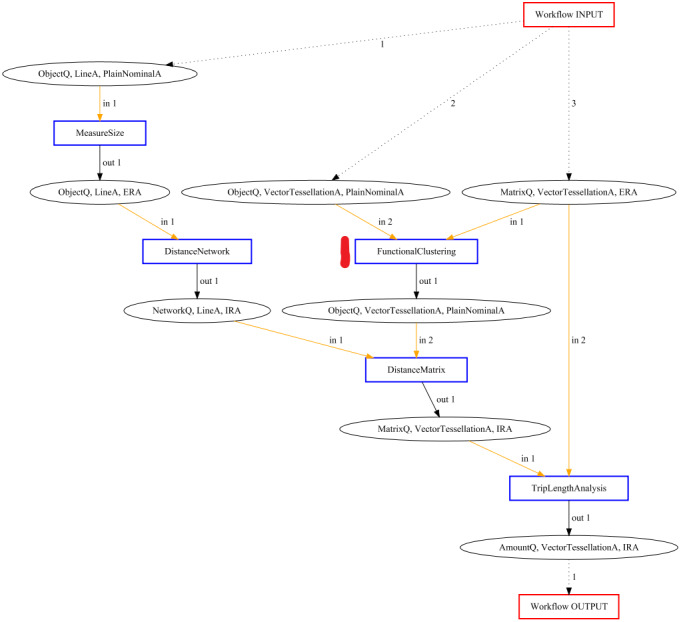
Example of a *redundancy error* produced by the CCD model for Task 7. The workflow produces a correct result of trip length analysis from roads (1), municipalities (2) and an interaction file (3), but an unnecessary functional clustering step is added

## ENDNOTES

1. SAT solvers, for example, form a basis for algorithms behind workflow synthesis (Lamprecht et al., 2010) and belong to symbolic AI.

2. This idea is rooted in a suggestion made by Werner Kuhn (cf. 2012) in personal conversation, so we can only partially take credit for it.

3. Web Ontology Language; see https://www.w3.org/OWL/.

4. Relations behave similarly to tables in a database or to entity relationship models in that they have primary and foreign keys. In the following, such analogy is used only to illustrate our idea, not to imply that core concepts are actually implemented as tables.

5. Extensivity in this case would need to take account of the *product* of origin and destination regions. Formalization of this kind of unary and binary extensivity is considered future work.

6. Think, for example, about grid or lattice graphs, which represent a network of raster cells or lattice polygons.

7. In the future we plan to extend the coverage of software to further network functions, such as the functions available in QGIS.

8. Detailed tasks descriptions can be found in Appendix A.

9. A more comprehensive documentation of the analysis is available at http://geographicknowledge.de/pdf/Flowmap%20Applications%20Q1‐16.pdf

10. Meaning that the workflow may still appear later in the process, but not within the first five workflows.

11. “Measure size”, for example, is implemented in Flowmap only as part of the network topology generation.

## APPENDIX A Specification of analytical tasks

### A.1

This appendix contains more detailed descriptions of the analytical tasks 1–12 for designing workflows, which were used to build and evaluate our conceptual model.

#### A1 Distance‐based network analysis

##### A1.1 Fan potential of municipalities

Starting from the number of inhabitants of municipalities, the potential of fans for a club in a municipality can be assessed by assuming a fixed distance threshold that these potential football fans would be willing to travel:

**Workflow Task 1** *”What is the potential number of football fans with a certain travel distance for each municipality in the Netherlands?”*

**Task Specification 1** input: (1) ROADS08, (2) POP_2003

goal types: OE (ObjectQ, RegionA, ERA)

use type: OIO* (MatrixQ, IRA)

For this task, we start with the roads file and the population data on municipalities, and the goal is to assess some object‐based extensive measure (the number of football fans reachable at some travel distance from a given municipality). To account for the concept of travel distances, we request in addition that some intensive matrix be used in the solution.

Alternatively, we can measure a minimum travel distance to reach a threshold number of fans that a football club can attract:

**Workflow Task 2** *”What is the potential minimal travel distance to reach a certain number of football fans for each municipality in the Netherlands?”*

**Task Specification 2** input: (1) ROADS08, (2) POP_2003

goal types: OI (ObjectQ, RegionA, IRA)

use types: OIO* (MatrixQ, RegionA, IRA)

Starting again with the roads file and the population data, our goal here is to estimate some object‐based intensive measure (minimal travel distance of some number of fans). For the same reason as above, we require that some distance matrix be used in the solution.

Both kinds of analysis result in a map that shows a potential for each municipality. Figure 1a shows the map for Task 2, in which all high‐ranking (red) municipalities are covered by at least one actual stadium. This supports the validity of the chosen potential measure.

##### A1.2 Accessibility of football clubs from municipalities

In this task, we are interested in finding out how accessible football clubs are for each municipality:

**Workflow Task 3** *”What is the accessibility of football clubs for each municipality in the Netherlands?”*

**Task Specification 3** input: (1) ROADS08, (2) MUNCLUB

goal types: OI (ObjectQ, RegionA, IRA)

use types: OIO* (MatrixQ, RegionA, IRA)

Here we start with plain municipalities (including some nominal attribute) and roads, to assess some object‐based intensive measure (the accessibility of football clubs). Since accessibility implies distance measurements, we likewise require that some distance matrix be used in the solution.

##### A1.3 Accessibility of football clubs from roads

Roads and intersections are the objects that constitute a road network. Here we determine the distance between each road and its closest football club.

**Workflow Task 4** *”What is the accessibility of football clubs from each road?”*

**Task Specification 4** input: (1) ROADS08, (2) MUNCLUB

goal types: OI (ObjectQ, LineA, IRA)

use types: OIO (NetworkQ, LineA, IRA)

Using roads and clubs as input, we request some object‐based intensive measure on lines, representing *road objects*. We require distances measured on some line network to account for the concept of accessibility from roads (Figure A1).

#### A2 Interaction‐based network analysis

##### A2.1 Numbers and flows of football fans

Flows in a matrix are shown in terms of the thickness of connecting lines in Figure A2a.

Starting from an interaction matrix, a simple transformation is needed in order to assess how many football fans originate in each municipality.

**Workflow Task 5** *”What is the number of season ticket holders for each municipality in the Netherlands?”*

**Task Specification 5** input: (1) STICKET2

goal types: OE (ObjectQ, RegionA, ERA)

In this task, we start from the interaction table with ticket holders and request some extensive object‐based measure (number of ticket holders). To generate the map in FigureA2b, we need to summarize the interaction table over one of its keys. This shows that season ticket holders seem concentrated around existing football clubs.

##### A2.2 Functional distance clustering of football clubs

**Workflow Task 6** *”To which functional cluster does a football club belong?”*

**Task Specification 6** input: (1) STICKET2, (2) MUNCLUB

goal types: OS, ON (ObjectQ, RegionA, PlainNominalA)

Here, we start again with the interaction table. Together with the plain municipality data, it should be used to derive some nominal attribute for objects (cluster labels for municipalities).

The dendrogram in Figure A3a shows the progress of the fusion process as residential municipalities are merged with football clubs. The map in Figure A3b shows, in purple, the fusion lines representing a merge between a residential municipality and a football club. The blue fusion lines indicate the first 24 merges between football clubs resulting in 14 (sub‐)regional clusters; After this stage over 86.6% of all ticket holders are internalized in one of the clusters. The red fusion lines indicate the next nine steps, after which five clusters at the national level remain.

##### A2.3 Football trip length distribution and trip end ranking

**Workflow Task 7** *”What is the average travel time to/rank of their football club if all ticket holders were to travel by car?”*

**Task Specification 7** input: (1) ROADS08, (2) MUNCLUB, (3) STICKET2,

goal types: I (IRA)

In this task we use roads, municipalities and an interaction table to assess some intensive measure, namely the mean travel time. Alternatively, one can also compare a given trip with potentially closer trip alternatives, by ranking destinations (football clubs) for each given origin (municipality) with respect to the closest destination. When we weight this rank by the amount of interaction and average it over all flows, we obtain an average rank number. This is called *trip end ranking*. In this example, it shows that out of a choice of 37 clubs the average season ticket holder chooses the 2.5th closest club.

##### A2.4 Trade area analysis of football clubs

**Workflow Task 8** *”What is the area enclosing 60% of the number of season ticket holders (trade area) closest to each football club in the Netherlands?”*

**Task Specification 8** input: (1) MUNCLUB, (2) ROADS08, (3) STICKET2

goal types: OS, ON (ObjectQ, RegionA, PlainNominalA)

use types: OEO* (MatrixQ, ERA), OIO* (MatrixQ, IRA)

In this task we are requesting some object‐based region enclosing a given number of the closest ticket holders. The term “closest” in this task implies the use of some distance matrix, and the number implies some extensive matrix between clubs and municipalities. We are interested in the size and the extent of overlap of these trade areas (Figure A4a). It can be seen that the big three clubs (Ajax Amsterdam, Feyenoord Rotterdam and PSV Eindhoven) fully dominate their neighbors.

#### A3 Flow generation

##### A3.1 Gravity model of football fan interaction

**Workflow Task 9** *”What is the potential number of season tickets to be sold in each municipality for each football club in the Netherlands, if some form of distance decay is assumed?”*

**Task Specification 9** input: (1) MUNCLUB, (2) ROADS08, (3) STICKET2

goal types: OEO* (MatrixQ, RegionA, ERA)

Given some road data, some ticket interaction data and some municipality/club data, we are interested in predicting an extensive matrix, denoting the numbers of tickets sold for a municipality and some club.

**Workflow Task 10** *”What is the attractiveness of football clubs for season ticket holders?”*

**Task Specification 10** input: (1) MUNCLUB, (2) ROADS08, (3) STICKET2

goal types: OI (ObjectQ, RegionA, IRA)

In this task, our goal is to assess some intensive object‐based measure (attractiveness of clubs) using the same input.

**Workflow Task 11** *”What is the potential number of season ticket holders for remaining football clubs, when the same distance decay effect and the same attractiveness for the remaining clubs are assumed as before closure?”*

**Task Specification 11** input: (1) MUNCLUB, (2) ROADS08, (3) STICKET2, (4) NEWCLUBS

goal types: OE (ObjectQ, RegionA, ERA)

In this task, we use some hypothetical club attractiveness together with roads and other data to obtain some object‐based quantities (ticket holders for each club).

**Task Specification 11a** input: (1) MUNCLUB, (2) ROADS08, (3) STICKET2

goal types: OE (ObjectQ, RegionA, ERA)

use types: OI (ObjectQ, RegionA, IRA)

Note that in Task 11a we leave out the fourth input, but additionally require some intermediate step that generates this input.

##### A3.2 Traffic load analysis for football trip flow

**Workflow Task 12** *”What would be the traffic load for each road in the Netherlands, assuming all season ticket holders were to travel by car at the same time?”*

**Task Specification 12** *Traffic load for each road in a network assuming shortest paths*:

input: (1) MUNCLUB, (2) STICKET2, (3) ROADS08

goal types: OEO (NetworkQ, LineA, ERA)

In this task we start with the interaction table, the municipalities and the roads to estimate some extensive (flow) measure on these roads. Based on flow assignment, we find that the traffic load caused by season ticket holders may run up to almost 49,000 on a single road segment in the vicinity of the most popular football clubs (Figure A2b).
